# Toxicological Properties of 7-Methylguanine, and Preliminary Data on its Anticancer Activity

**DOI:** 10.3389/fphar.2022.842316

**Published:** 2022-07-06

**Authors:** Kirill Kirsanov, Timur Fetisov, Elena Antoshina, Lubov Trukhanova, Tatiana Gor’kova, Olga Vlasova, Irina Khitrovo, Ekaterina Lesovaya, Nataliya Kulbachevskaya, Tatiana Shcherbakova, Gennady Belitsky, Marianna Yakubovskaya, Vytas Švedas, Dmitry Nilov

**Affiliations:** ^1^ Blokhin Cancer Research Center, Moscow, Russia; ^2^ Peoples’ Friendship University of Russia, Moscow, Russia; ^3^ Pavlov Ryazan State Medical University, Ryazan, Russia; ^4^ Belozersky Institute of Physicochemical Biology, Lomonosov Moscow State University, Moscow, Russia; ^5^ Faculty of Bioengineering and Bioinformatics, Lomonosov Moscow State University, Moscow, Russia

**Keywords:** 7-Methylguanine, inhibitor, cancer, carcinogenicity, toxicity

## Abstract

7-Methylguanine (7-MG) competitively inhibits the DNA repair enzyme poly(ADP-ribose) polymerase (PARP) and RNA-modifying enzyme tRNA-guanine transglycosylase (TGT) and represents a potential anticancer drug candidate. Furthermore, as a natural compound, it could escape the serious side effects characteristic for approved synthetic PARP inhibitors. Here we present a comprehensive study of toxicological and carcinogenic properties of 7-MG. It was demonstrated that 7-MG does not induce mutations or structural chromosomal abnormalities, and has no blastomogenic activity. A treatment regimen with 7-MG has been established in mice (50 mg/kg per os, 3 times per week), exerting no adverse effects or changes in morphology. Preliminary data on the 7-MG anticancer activity obtained on transplantable tumor models support our conclusions that 7-MG can become a promising new component of chemotherapy.

## 1 Introduction

7-Methylguanine (7-MG) (Shapiro et al., 1968) is a degradation product of nucleic acids which is present in small amounts in human urine (Bromberg et al., 1957a; Bromberg et al., 1957b; Lothrop and Uziel, 1983; Svoboda and Kasai, 2004; Rodríguez-Gonzalo et al., 2013; Raćkowska et al., 2019) and may be considered an indicator of whole-body RNA turnover (Sander et al., 1986a; Sander et al., 1986b; Sander et al., 1986c; Topp et al., 1987). In mRNA the guanosine cap is methylated due to methyltransferase activity, that is required for maturation and translation (Shuman, 2002; Shafer et al., 2005; Topisirovic et al., 2011; Varshney et al., 2016). Furthermore, 7-MG adducts are normally present in DNA, exposed to various exogenous and endogenous methylating agents, and their number is increasing on aging (Park and Ames, 1988; Tan et al., 1990; Mustonen and Hemminki, 1992; O'Connor, 1993; Tamae et al., 2009). There is no evidence, however, for synthesis of nucleotides from free 7-MG base or for its direct incorporation into nucleic acids (Craddock et al., 1968; Kaina et al., 1983; Kerr, 1985; Kerr, 1990). A certain proportion of 7-MG is converted to 8-hydroxy-7-methylguanine by xanthine oxidase or demethylated (Weissmann and Gutman, 1957; Borowitz et al., 1965; Litwack and Weissmann, 1966; Skupp and Ayvazian, 1969).

Recently we have shown that 7-MG inhibits DNA repair enzymes poly(ADP-ribose) polymerases, PARP1 and PARP2, in a competitive manner and accelerates apoptotic death of cancer cells induced by cisplatin and doxorubicin (Nilov et al., 2016; Nilov et al., 2018a; Nilov et al., 2020a). These PARP enzymes bind to DNA breaks and synthesize a signal polymer poly (ADP-ribose) from NAD^+^ molecules to activate the excision repair proteins (Hassler and Ladurner, 2012; Drenichev and Mikhailov, 2015; Ray Chaudhuri and Nussenzweig, 2017; Alemasova and Lavrik, 2019; Nilov et al., 2020b). Inhibitors of PARP1/2, therefore, can exert anti-proliferative effect and be combined with DNA damaging agents (Cepeda et al., 2006; Martin et al., 2008; Ferraris, 2010; Lord et al., 2015; Nilov et al., 2018b). We have demonstrated that 7-MG forms substrate-specific interactions with the Gly863 and Tyr907 residues in the PARP1/2 active site and suppresses DNA-dependent PARP activity in three different assays (biochemical assay with radiolabeled NAD^+^, fluorescence anisotropy assay, and Förster resonance energy transfer microscopy assay with nucleosome particles) (Nilov et al., 2016; Nilov et al., 2020a). This results in the formation of nonproductive PARP–nucleosome complexes and likely prevents further steps in DNA repair, replication and transcription, leading to cancer cell death (Maluchenko et al., 2019; Nilov et al., 2020a). 7-MG is also known as a competitive inhibitor of RNA-modifying enzyme tRNA-guanine transglycosylase (TGT) which substitutes the guanine base with 7-deazaguanine derivative queuine (Farkas et al., 1984; Johannsson et al., 2018). In a recent paper, it was shown that TGT deficiency could significantly suppress the proliferation and migration of cancer cells (Zhang et al., 2020). From the point of view of polypharmacology, such a multitarget (PARP1/2, TGT) mechanism of a drug candidate may be promising, if adverse effects are negligible (Bolognesi, 2013; Medina-Franco et al., 2013).

FDA-approved synthetic PARP1/2 inhibitors olaparib, rucaparib, niraparib (Frampton, 2015; Mittica et al., 2018; Zimmer et al., 2018) can cause side effects likely related to the nonselective interaction with numerous NAD^+^-binding proteins and nonspecific effects on the organism. Myelodysplastic syndrome/acute myeloid leukemia occurred in some patients after treatment with above-mentioned synthetic inhibitors, and some cases were fatal (Malyuchenko et al., 2015; Sonnenblick et al., 2015; Wang et al., 2016; Ohmoto and Yachida, 2017; Jain and Patel, 2019). 7-MG, being a natural compound, may have a more favorable toxicity profile, which is also supported by QSAR modeling (Nilov et al., 2016; Nilov et al., 2018a). In this article, we present the results of a comprehensive experimental study of toxicological and carcinogenic properties of 7-MG that establish the basis for further testing of its anticancer activity.

## 2 Materials and Methods

### 2.1 Toxicology Studies

**FIGURE 1:**
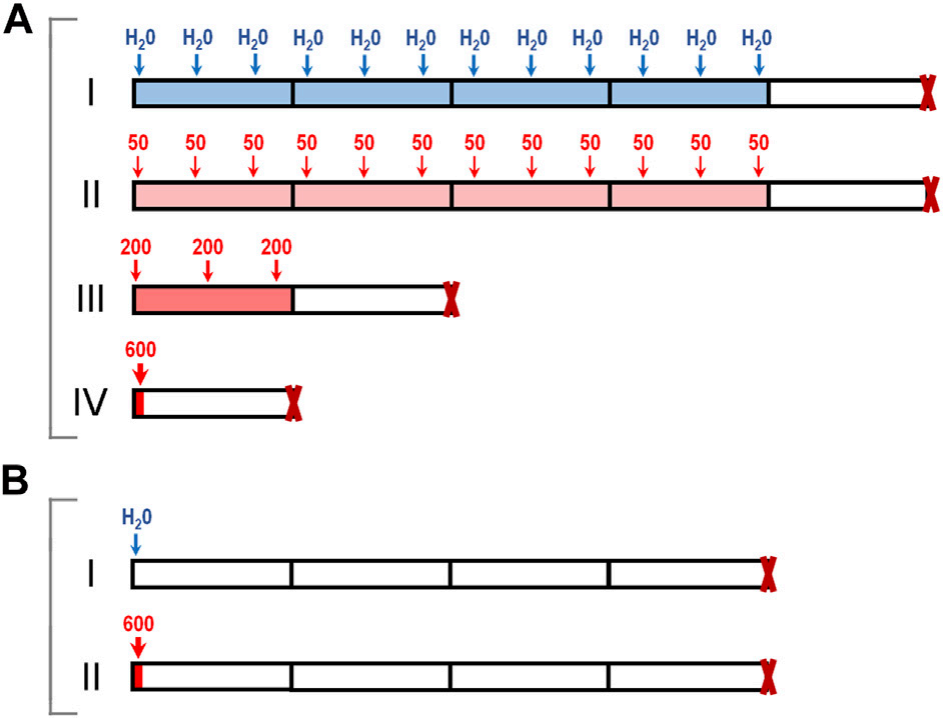
Toxicology study of 7-MG: schematic representation of experiments. **(A)** CBA mice were treated either with water (group I) or with single and multiple doses of 7-MG (groups II–IV, total dose for each group was 600 mg/kg); **(B)** CBA, BALB/c, or C57BL/6 mice were treated with water (groups I) or a single 600 mg/kg dose of 7-MG (group II), and euthanized 4 weeks later.

Six-week-old female CBA, BALB/c, and C57BL/6 mice were obtained from the Stolbovaya farm of the Federal Medical Biological Agency (http://www.scbmt.ru). These mouse strains are widely used in toxicology studies and in studies involving transplantable tumor models. 7-MG (Sigma-Aldrich, product No. 67073) was administered orally by gavage in experiments A and B (Figure 1). In experiment A, CBA mice were divided randomly to four treatment groups of 10 animals: control group I, drinking water (3 times per week for 4 weeks); group II, 50 mg/kg 7-MG (3 times per week for 4 weeks); group III, 200 mg/kg 7-MG (3 times per week for 1 week); group IV, 600 mg/kg 7-MG (single dose). Animals were euthanized by cervical dislocation 1 week after the last treatment. In experiment B, mice of each strain (CBA, BALB/c, C57BL/6) were divided into two groups, group I (drinking water) and group II (600 mg/kg 7-MG), and euthanized 4 weeks after single-dose administration. Lungs, heart, liver, spleen, thymus, kidneys, adrenal glands, pancreas, stomach, small and large intestines were collected from euthanized mice and inspected. The tissues were processed for light microscopy by fixing in 10% buffered formalin, dehydrating, and embedding in paraffin. Histological analysis was performed on sections stained by hematoxylin-eosin. Organ lesions were detected on the examined sections (representative microphotographs of the found abnormalities are shown in Supplementary Figure S1), and the number of lesions per animal was counted.

### 2.2 Carcinogenicity Studies

The Ames test was performed in *S. typhimurium* strains TA98 and TA100 as described previously (Maron and Ames, 1983). Three 7-MG doses (1.4, 7.0, and 35.0 µg/plate) were tested with and without rat liver S9 fraction. Distilled water was used as a negative control; 3,4-benzopyrene (4.4 µg/plate), 2-acetylaminofluorene (22.0 µg/plate), 4,9-diazapyrene derivative (8.8 µg/plate), and sodium azide (8.8 µg/plate) served as positive controls. Plates were incubated for 72 h and then revertant colonies were counted. The comet assay was performed as described previously (Singh et al., 1988). Immortalized human kidney epithelial cells (NKE-hTERT) were treated with different concentrations of 7-MG (0.02 and 0.2 mg/ml) for 24 h and then embedded in agarose on microscope slides. After cell lysis and electrophoresis, slides were stained with DNA dye (Vista Green) and the number of comets was counted. Distilled water was used as a negative control and cisplatin (25 µg/ml) served as a positive control. The somatic mutation and recombination test in *D. melanogaster* was based on previous work (Kirsanov et al., 2011). Five wild type males and 10 *wts* ^*P4*^/*TM3* females were placed into vials for breeding. Heterozygous larvae were treated with 7-MG (1 and 2 mg/vial); distilled water and oxoplatin (0.2 mg/vial) were used as controls. Adult F1 males and females were examined for the presence of tumors using a binocular microscope.

The chromosomal aberration assay was performed using standard procedure (Albertini et al., 2000). C57BL/6 mice were divided to groups of 5 animals and treated with 7-MG (50–250 mg/kg per os). Distilled water was used as a negative control and cyclophosphamide (50 mg/kg i.p.) served as a positive control. Bone marrow cells were collected 24 h after the treatment; to arrest proliferating cells at metaphase, animals received colchicine (0.004% i.p.) 3 h prior to euthanasia. Cells were obtained from the femurs, stained on slides with Giemsa, and analyzed by microscopy.

### 2.3 Anticancer Activity

Six-week-old female CBA mice were obtained from the Stolbovaya farm. Uterine sarcoma US-322 and cervical squamous cell carcinoma RShM-5 (originally derived from CBA mice exposed to 1,2-dimethylhydrazine and 3-methylcholanthrene, respectively) (Treshalina et al., 2000; Turusov et al., 2005; Bunyatyan et al., 2019) were inoculated subcutaneously by injecting 0.5 ml of tumor cell suspension (0.1 g/ml in PBS) into the right axillary cavity. Mice with US-322 were divided into three treatment groups of 10 animals: control group I, PBS (s.c., 3 times per week); group II, 7-MG (50 mg/kg per os, 3 times per week); group III, cisplatin (2.5 mg/kg s.c., 2 times within a week after inoculation). Mice with RShM-5 were divided into five groups of 10 animals: group I, PBS (s.c., 3 times per week); group II, 7-MG (50 mg/kg per os, 3 times per week); group III, 7-MG for 1 week (50 mg/kg per os, 3 times within a week after inoculation); group IV, cisplatin (1.5 mg/kg s.c., 3 times within a week after inoculation); group V, cisplatin + 7-MG (1.5 mg/kg s.c. + 50 mg/kg per os, 3 times within a week after inoculation). For the combined treatment, 7-MG was administered 3 h prior to cisplatin. The length and width of a subcutaneous tumor were measured with a digital caliper, and the tumor volume was calculated as 1/2 (length×width^2^).

The animal protocols were approved by the Local Committee for Ethics of Animal Experimentation (Blokhin Cancer Research Center, decision 2019-5 dated 11 February 2019), experiments were conducted in accordance with resolution 81 of the Eurasian Economic Commission and directive 2010/63/EU (on the protection of animals used for scientific purposes).

### 2.4 Statistical Analysis

Statistical significance of the difference between animal groups was assessed with the Pearson’s chi-squared test (study of anticancer activity, study of chromosomal abnormalities in mice), Student’s *t*-test (analysis of organ weights), and Fisher’s exact test (study of blastomogenic activity in flies). Significant differences between cells in the comet assay were assessed with the Fisher’s exact test. Data processing was carried out using the Statistica software (StatSoft Inc.).

## 3 Results

### 3.1 Toxicology Studies

The adverse effects of 7-MG that can result either from a single or multiple exposures were assessed using four groups of female CBA mice, as presented in Figure 1A. A maximum dose of 600 mg/kg was chosen based on mouse/rat oral LD_50_ values (40–500 mg/kg) predicted for 7-MG with QSAR software ACD/Percepta (www.acdlabs.com). Oral administration of 7-MG to group II (50 mg/kg, 3 times per week for 4 weeks), group III (200 mg/kg, 3 times per week for 1 week), and group IV (600 mg/kg, single dose) was not lethal to any of the animals. Visual observation revealed no apparent lesions or abnormalities of internal organs in treated mice. However, a significant elevation in spleen weight was produced in group IV (Supplementary Table S1). Histological analysis of the heart, thymus, kidney, adrenal gland, pancreas and stomach showed no abnormalities in all treatment groups. Lung, liver, and spleen tissues were affected only in group IV (Table 1). In this group, small lymphoid infiltrates developed in the lungs and liver (Supplementary Figures S1A,B). The splenic white pulp had poorly defined follicles and lacked germinal centers, and the red pulp was infiltrated with lymphoid cells (Supplementary Figure S1C). In addition, focal lymphoid hyperplasia of the small and large intestines was found in groups III and IV (Supplementary Figure S1D).

**TABLE 1:** Histological abnormalities of internal organs (+) in CBA mice treated with 7-MG.

Group	Lungs	Liver	Spleen	Intestine
I control	—	—	—	—
II 50 mg/kg^a^	—	—	—	—
III 200 mg/kg^a^	—	—	—	+
IV 600 mg/kg	+	+	+	+

a Multiple-dose administration.

The stimulating effect on lymphoid tissue caused by a single 600 mg/kg dose of 7-MG was then investigated in more detail using different mouse strains: CBA, BALB/c and C57BL/6. Animals were administered with either water or 7-MG (600 mg/kg), and samples of lung, liver, spleen, and intestine were collected after 4 weeks (Figure 1B). Microscopic examination revealed the persistence of lymphoid lesions produced by a high dose of 7-MG in all strains. Lung, liver and intestine abnormalities were found in nearly all animals (Supplementary Table S2), and the splenic microarchitecture was affected in 33% of CBA mice, 30% of BALB/c, and 60% of C57BL/6. However, 7-MG treatment was not lethal to any of the animals and had no significant effect on body weight (Supplementary Figure S2). The median lethal dose (LD_50_) is therefore expected to be substantially greater than 600 mg/kg, which allows us to classify 7-MG as only slightly toxic inhibitor.

These results lead to a conclusion that 50 mg/kg administration 3 times per week may be an optimal regimen, which is devoid of adverse effects and can be readily applied in further testing for anticancer activity of 7-MG in mice (see the Section 3.3).

### 3.2 Carcinogenicity Studies

**FIGURE 2:**
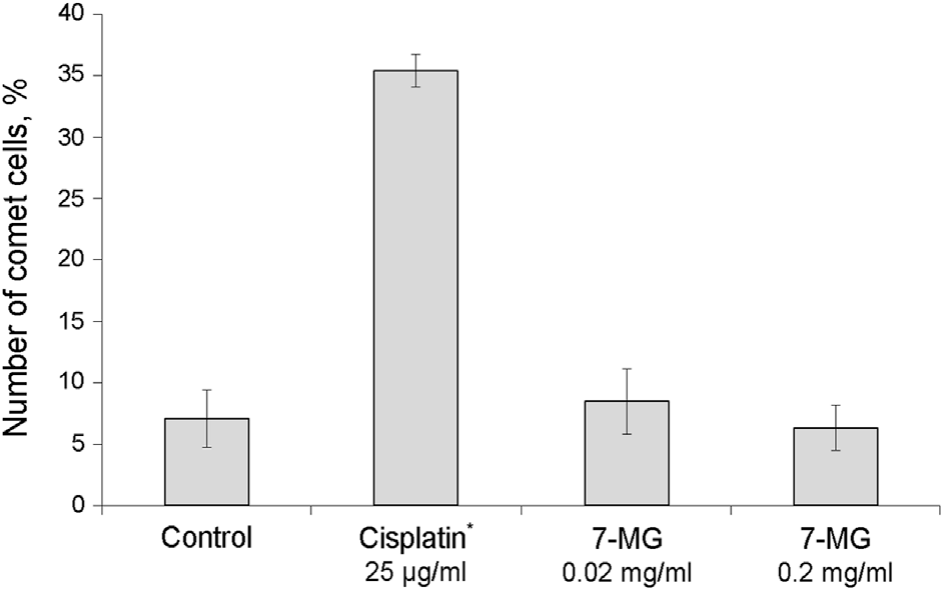
Study of the ability of 7-MG to produce DNA strand breaks in immortalized human kidney epithelial cells using the comet assay (500 cells were analyzed per slide). *Significant difference from the control cells (Fisher’s exact test, *p* < 0.05).

The mutagenic and carcinogenic properties of 7-MG have been studied using various short term tests: the Ames test (uses bacterial strains to assess the mutagenic potential) (Maron and Ames, 1983), comet assay (detects DNA strand breaks at the level of the individual cell) (Collins, 2004), chromosomal aberration assay (detects structural chromosomal abnormalities in mice) (Albertini et al., 2000), and somatic mutation and recombination test (uses *Drosophila melanogaster* to assess the mutagenic, recombinogenic and blastomogenic potential) (Sidorov et al., 2001). The mutagenicity of 7-MG was tested in *Salmonella typhimurium* strains, TA98 and TA100, both with and without metabolic activation by rat liver S9 fraction. It was demonstrated that 7-MG does not induce frameshift mutations or base-pair substitutions (Table 2). The comet assay showed that 7-MG does not produce DNA damage in immortalized human kidney epithelial cells (Figure 2; Supplementary Figure S3), and the chromosomal aberration assay showed that it does not induce chromatid or chromosome breaks in bone marrow cells of C57BL/6 mice (Table 3; Supplementary Figure S4). The somatic mutation and recombination test detected no blastomogenic activity of 7-MG in *wts*/*+* heterozygotes of *D. melanogaster* (Table 4).

**TABLE 2:** Study of the mutagenicity of 7-MG in *S. typhimurium* strains TA98 (detects frameshift mutagens) and TA100 (detects mutagens that cause base-pair substitutions).

Tested compound	Dose, µg/plate	TA98				TA100			
		−S9		+S9		−S9		+S9	
		M	MA	M	MA	M	MA	M	MA
Control	—	9 ± 1	—	16 ± 5	—	43 ± 9	—	53 ± 2	—
BP	4.4			139 ± 15	+			623 ± 23	+
AAF	22.0			448 ± 53	+			433 ± 33	+
DP	8.8	248 ± 42	+						
AZ	8.8					397 ± 31	+		
7-MG	1.4	11 ± 3	—	16 ± 0.4	—	45 ± 1	—	56 ± 3	—
	7.0	10 ± 1	—	14 ± 3	—	35 ± 8	—	44 ± 8	—
	35.0	11 ± 1	—	14 ± 2	—	41 ± 3	—	56 ± 2	—

7-MG, 3,4-benzopyrene (BP), 2-acetylaminofluorene (AAF), 4,9-diazapyrene derivative (DP), and sodium azide (AZ) were tested with and without rat liver S9 fraction. The number of revertant colonies (M) was counted to assess the mutagenic activity (MA). MA was considered positive if M in the treated plates exceeded that in the control more than twice.

**TABLE 3:** Study of the ability of 7-MG to induce chromosomal aberrations in C57BL/6 mice.

Group		Dose, mg/kg	Number of damaged cells per 500 cells			
			Chromatid breaks	Chromosome breaks	Multiple aberrations	Total number
1 day	I control, males	—	8	0	0	8
	II cyclophosphamide, males	50	30	1	11	42^a^
	III 7-MG, males	50	7	0	0	7
	IV 7-MG, males	250	7	0	0	7
5 days	V control, males	—	9	0	0	9
	VI control, females	—	10	0	0	10
	VII 7-MG, males	5 × 50^b^	7	0	0	7
	VIII 7-MG, females	5 × 50	9	0	0	9

Bone marrow cells were collected 24 h after the last treatment.

a Significant difference from the control group (Pearson’s chi-squared test, *p* < 0.01).

b 50 mg/kg per day.

**TABLE 4:** Study of the blastomogenic activity of 7-MG in *D. melanogaster*.

Tested compound	Dose, mg/vial	Number of flies	Number of tumors	Tumor frequency, %
Control	—	452	14	3.1
Oxoplatin	0.2	564	128	22.7^a^
7-MG	1.0	488	14	2.9
	2.0	405	9	2.2

a Significant difference from the control (Fisher’s exact test, *p* < 0.01).

### 3.3 Anticancer Activity

In previous sections, the following findings were made: 1) 7-MG is not carcinogenic and 2) it can be safely administered in an appropriate dosage. As molecular mechanisms of 7-MG are known (PARP1/2 and TGT inhibition), a thorough investigation of its anticancer properties could be initiated *in vivo*, involving various transplantable tumor models and a set of existing drugs as active controls. Below are two illustrative examples demonstrating the utility of 7-MG as a component of chemotherapy.

**FIGURE 3:**
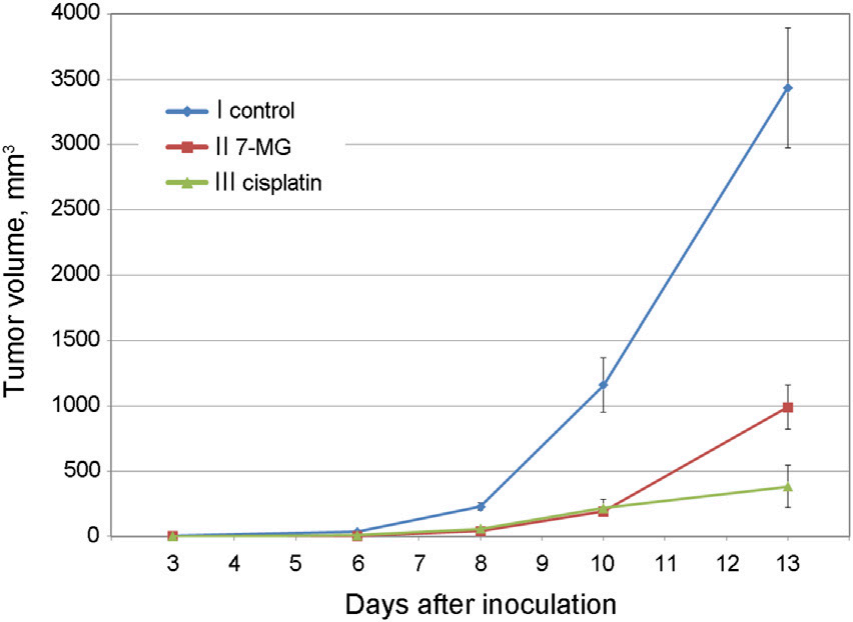
Dynamics of US-322 tumor growth in female CBA mice at different treatment regimens.

**FIGURE 4:**
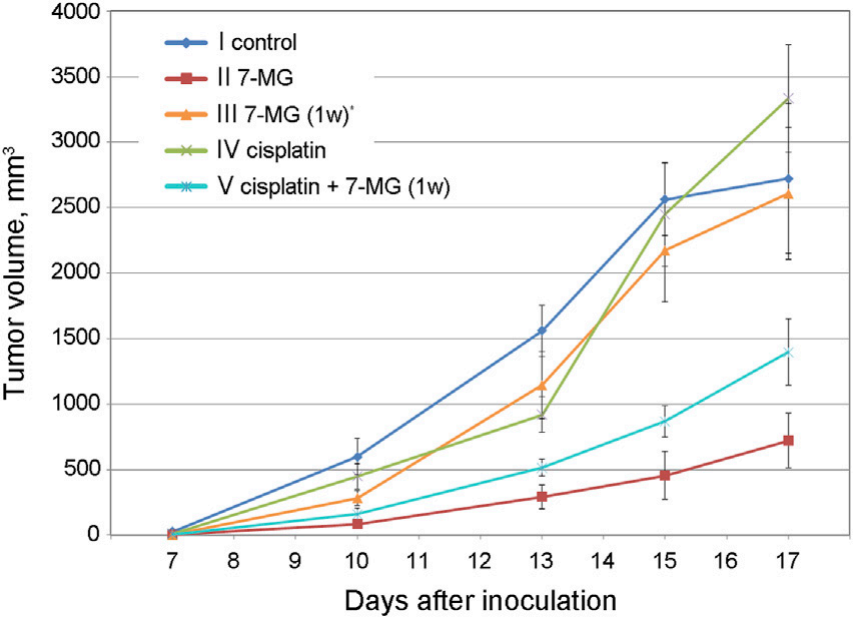
Dynamics of RShM-5 tumor growth in female CBA mice at different treatment regimens. *7-MG was given for 1 week only.

Preliminary studies of 7-MG anticancer activity at a safe dose were carried out using mouse transplantable tumor models of uterine sarcoma US-322 (Figure 3, Supplementary Table S3) and cervical cancer RShM-5 (Figure 4, Supplementary Table S4). Tumor nodes appeared in all control group animals within 3 and 7 days after inoculation, respectively. The US-322 model demonstrated a statistically significant inhibition of tumor growth by the treatment with 7-MG, 50 mg/kg 3 times per week (Figure 3, group II), and the effect of 7-MG was comparable to cisplatin (group III). In the case of RShM-5 model, an ineffective dose of cisplatin was used to test the ability of 7-MG to sensitize the tumor to DNA-damaging agents. It was revealed that the combined treatment with cisplatin and 7-MG within a week after inoculation inhibits tumor growth, whilst 7-MG or cisplatin administration alone exerted no significant effect (Figure 4, groups III–V). It is noteworthy that single-agent 7-MG treatment extended to more than 1 week (group II) had an even more pronounced antitumor effect compared to 1 week of combined treatment. Furthermore, group III (7-MG given for 1 week) clearly demonstrates that interruption of the 7-MG treatment results in accelerated tumor growth starting from the 10th day.

## 4 Discussion

PARP1/2 inhibitors represent a novel class of anticancer agents. Although initially proposed for treatment of BRCA-deficient tumors in women (breast or ovarian cancer), these inhibitors have also demonstrated efficacy in other models such as prostate and gastric cancers (Underhill et al., 2011; Sachdev et al., 2019). Soft tissue sarcomas were also shown to be sensitive to PARP inhibition combined with genotoxic chemotherapy (Ordóñez et al., 2015). The strong PARP1/2 suppression seems to be inherently toxic due to an important role played by these proteins in the organism, but attempts are continuing to find the proper balance between efficacy and toxicity of inhibitors. Fatigue, anemia, nausea and neutropenia together with a risk of myelodysplastic syndrome/acute myeloid leukemia accompany the use of synthetic PARP1/2 inhibitors (Walsh, 2018). The recently described inhibitor 7-MG is a natural nitrogenous base that could escape the serious side effects and become a promising new component of chemotherapy. Additionally, 7-MG inhibits the RNA-modifying enzyme TGT, which may enhance its anticancer activity.

The primary aim of this research was to outline the safety profile of 7-MG *in vivo*. We have established an oral regimen for 7-MG treatment in CBA mice (50 mg/kg, 3 times per week for up to 4 weeks) that exerts no adverse effects or changes in morphology. Adverse events were detected only at a maximum single dose of 600 mg/kg, in the form of small lymphoid infiltrates of non-inflammatory origin in the lungs and liver. These lesions may be resulted from the excessive inhibition of PARP (Beneke and Möröy, 2001; Ricks et al., 2015; Dasa et al., 2018) at concentrations much higher than therapeutic levels. The safety of 7-MG was also confirmed by the examination in four short-term carcinogenicity assays where it showed no mutagenic or blastomogenic effects.

Preliminary data obtained on mouse transplantable tumor models (uterine sarcoma, cervical carcinoma) demonstrated that 7-MG significantly reduces tumor growth at a safe dose and can also potentiate the activity of cisplatin. The interruption of the 7-MG treatment results in accelerated tumor growth, which highlights the advantages of a multiple-dose regimen. The molecular mechanism of 7-MG is fundamentally different from that of drugs like cisplatin. It acts by modulating the enzyme activity instead of causing DNA damage, and the multiple exposure to 7-MG is needed for the effective modulation. The present study of the natural 7-MG compound has confirmed its safety and potential tumor suppressing activity in mice. For further development, it would be important to identify the most sensitive tumors for the 7-MG treatment as well as to select DNA-damaging agents for the combination treatment.

## Data Availability

The original contributions presented in the study are included in the article/Supplementary Material, further inquiries can be directed to the corresponding author.

## Abbreviations

7-MG, 7-methylguanine; PARP, poly(ADP-ribose) polymerase; TGT, tRNA-guanine transglycosylase.
