# Supplementary material for: Toxicological Properties of 7-Methylguanine, and Preliminary Data on its Anticancer Activity
Source: Front Pharmacol. 2022 Jul 6;13:842316. doi: 10.3389/fphar.2022.842316 (PMC9299380; doi:10.3389/fphar.2022.842316)
Supplement: Supplementary file 1 [file DataSheet1.docx]

Supplementary Material


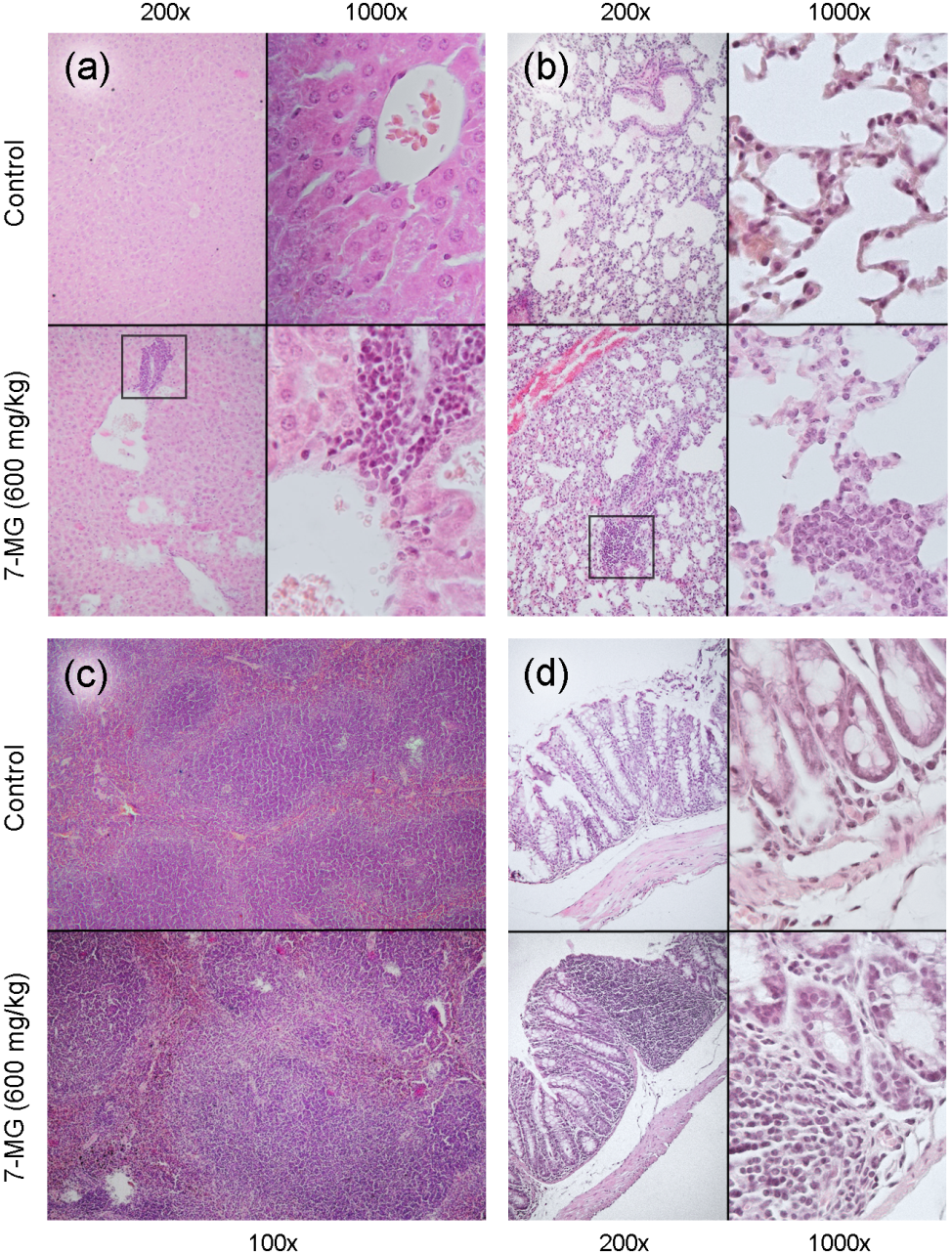


**Figure S1.** Histological abnormalities of internal organs in CBA mice treated with a single 600 mg/kg dose of 7-MG. (**a**) Lymphoid infiltrates in the liver; (**b**) Lymphoid infiltrates in the lungs; (**c**) Abnormal microarchitecture of the spleen; (**d**) Lymphoid hyperplasia of the large intestine.

**
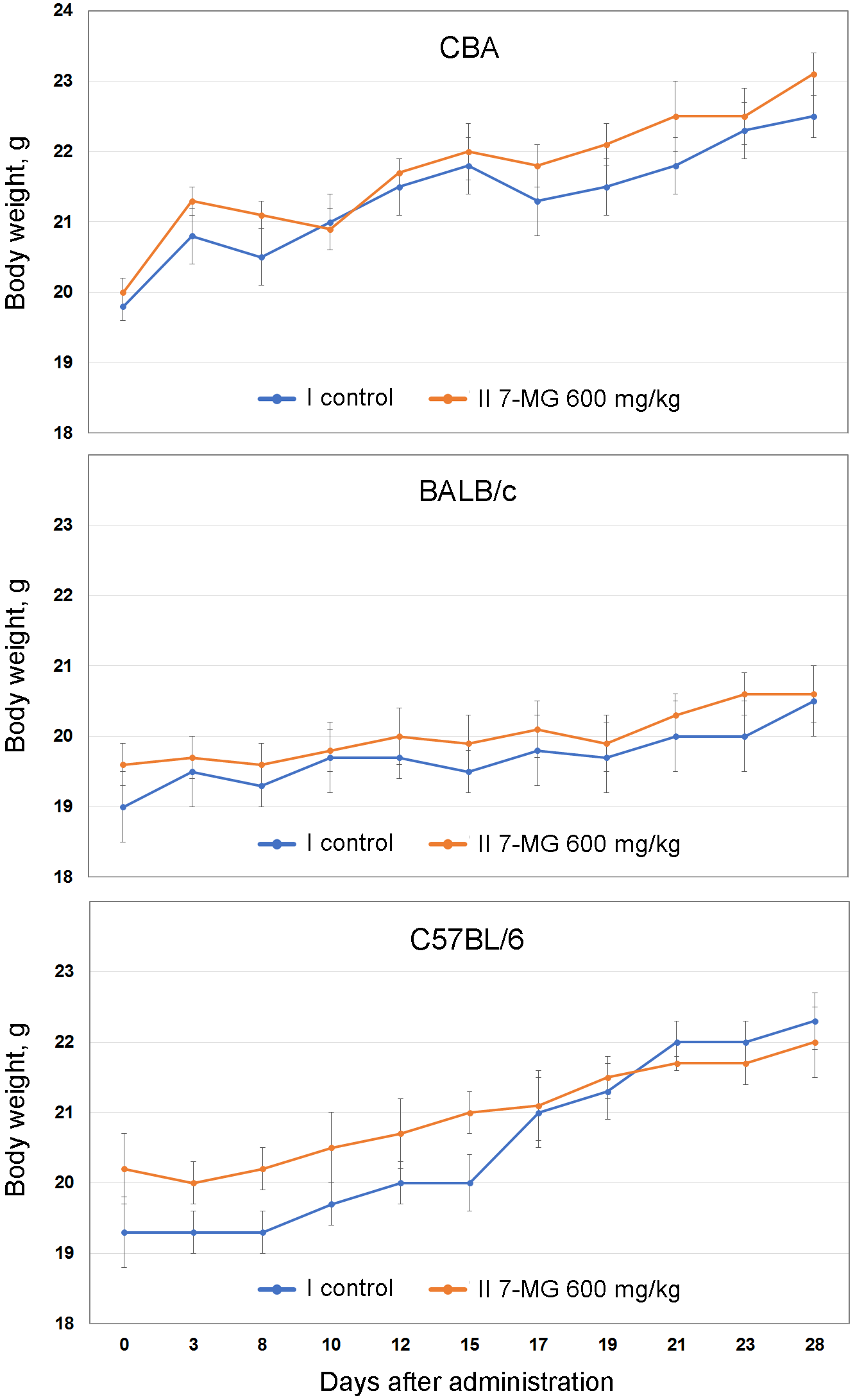
**

**Figure S2.** Body weights of female CBA, BALB/c and C57BL/6 mice treated with a single 600 mg/kg dose of 7-MG.

**
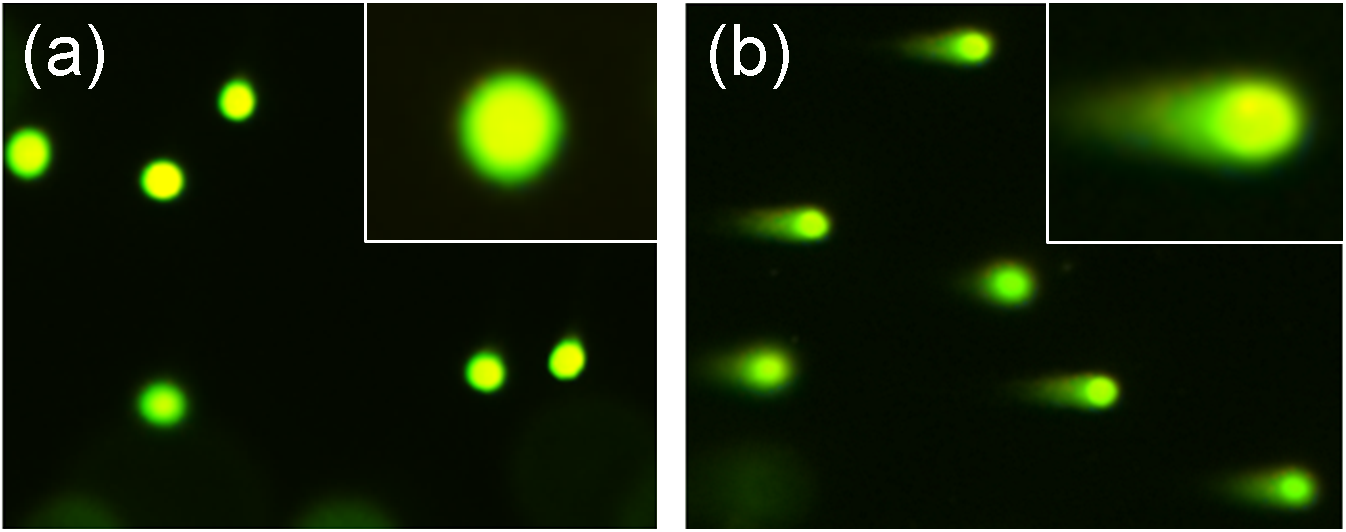
**

**Figure S3.** Representative images of the comet assay with immortalized human kidney epithelial cells. (**a**) Normal cells; (**b**) Comet cells (treated with cisplatin).

**
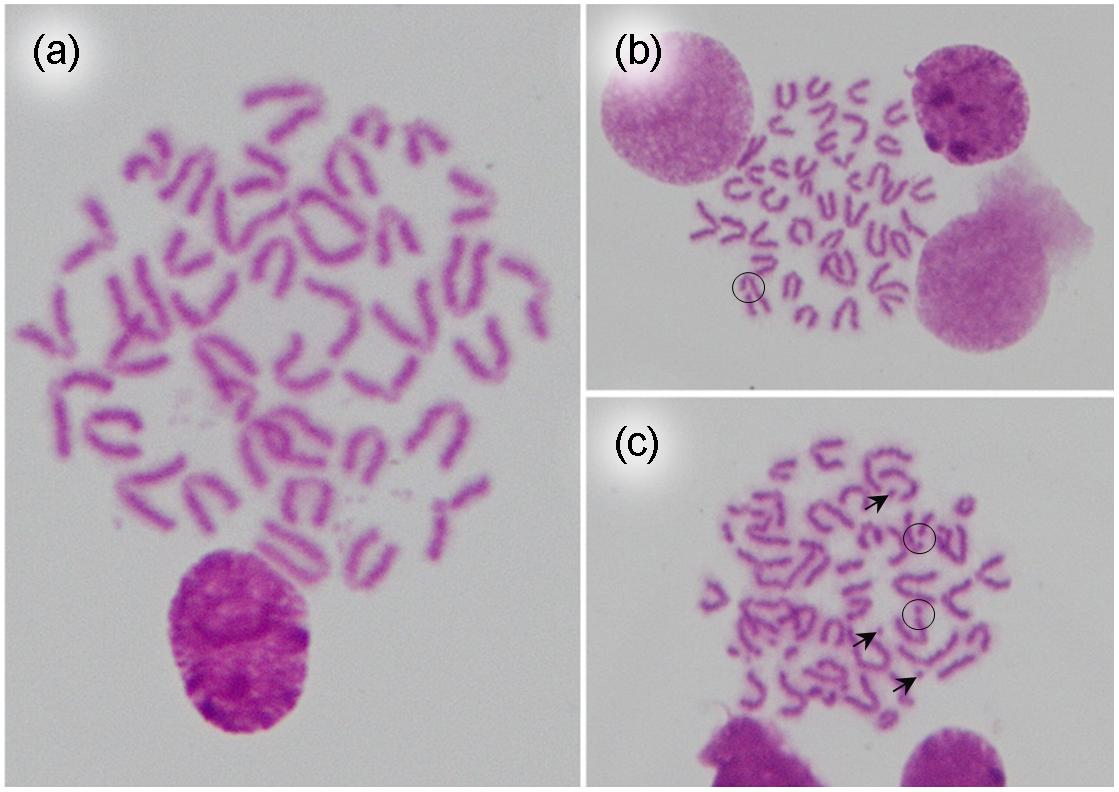
**

**Figure S4.** Representative images of the chromosomal aberration assay with bone marrow cells of C57BL/6 mice. (**a**) Normal cell; (**b**) Cell with a chromatid break marked with a circle. (**c**) Cell with multiple aberrations (chromatid breaks + chromatid fragments).

**Table S1.** Organ weight as a percentage of body weight in female CBA mice treated with 7-MG. *Multiple-dose administration. **Significant difference from the control group (Student’s *t*-test, p < 0.05).

| Group | Liver | Spleen | Thymus |
| --- | --- | --- | --- |
| I control | 5.42 ± 0.13 | 0.32 ± 0.01 | 0.25 ± 0.01 |
| II 50 mg/kg^*^ | 5.69 ± 0.13 | 0.35 ± 0.01 | 0.24 ± 0.01 |
| III 200 mg/kg^*^ | 5.95 ± 0.23 | 0.42 ± 0.09 | 0.21 ± 0.01 |
| IV 600 mg/kg | 6.78 ± 0.20 | 0.73 ± 0.07** | 0.24 ± 0.02 |

**Table S2.** Multiplicity of lesions of internal organs in female CBA, BALB/c and C57BL/6 mice treated with a single 600 mg/kg dose of 7-MG.

| Mouse strain | Number of lesions per animal | | |
| --- | --- | --- | --- |
|  | Lungs | Liver | Large intestine |
| CBA | 25.9 ± 3.1 | 14.6 ± 2.1 | 2.1 ± 0.5 |
| BALB/c | 10.4 ± 2.4 | 12.5 ± 2.4 | 3.3 ± 0.6 |
| C57BL/6 | 23.4 ± 4.2 | 24.2 ± 2.7 | 1.8 ± 0.4 |

**Table S3.** Dynamics of US-322 tumor growth in female CBA mice at different treatment regimens. *Statistical significance of the difference between groups was assessed with the Pearson’s chi-squared test.

|  | Days after tumor inoculation | | | | |
| --- | --- | --- | --- | --- | --- |
|  | 3 | 6 | 8 | 10 | 13 |
| Group | Tumor volume, mm^3^ (mean ± SEM) | | | | |
| I control | 3±1 | 36±8 | 228±29 | 1160±208 | 3435±460 |
| II 7-MG | 1±1 | 4±2 | 41±12 | 191±34 | 991±169 |
| III cisplatin | 2±1 | 9±2 | 58±11 | 218±66 | 383±162 |
| p I,II* | - | < 0.01 | < 0.01 | < 0.01 | < 0.01 |
| p I,III | - | < 0.01 | < 0.01 | < 0.01 | < 0.01 |
| p II,III | - | - | - | - | < 0.05 |

**Table S4.** Dynamics of RShM-5 tumor growth in female CBA mice at different treatment regimens. *7-MG was given for 1 week only. **Statistical significance of the difference between groups was assessed with the Pearson’s chi-squared test.

|  | Days after tumor inoculation | | | | |
| --- | --- | --- | --- | --- | --- |
|  | 7 | 10 | 13 | 15 | 17 |
| Group | Tumor volume, mm^3^ (mean ± SEM) | | | | |
| I control | 26±8 | 598±137 | 1560±195 | 2563±278 | 2722±572 |
| II 7-MG | 2±1 | 85±30 | 292±91 | 454±182 | 720±209 |
| III 7-MG (1w)* | 4±1 | 282±57 | 1145±254 | 2174±392 | 2608±506 |
| IV cisplatin | 11±3 | 449±97 | 918±136 | 2449±396 | 3334±409 |
| V cisplatin +  7-MG (1w) | 7±3 | 161±44 | 515±67 | 869±120 | 1396±254 |
| p I,II** | <0.01 | <0.01 | <0.01 | <0.01 | <0.01 |
| p I,III | <0.05 | <0.05 | - | - | - |
| p I,IV | - | - | <0.05 | - | - |
| p I,V | <0.05 | <0.01 | <0.01 | <0.01 | <0.05 |
